# Controlling nutritional status (CONUT) score as a preoperative risk assessment index for older patients with colorectal cancer

**DOI:** 10.1186/s12885-019-6218-8

**Published:** 2019-11-06

**Authors:** Yuka Ahiko, Dai Shida, Tomoko Horie, Taro Tanabe, Yasuyuki Takamizawa, Ryohei Sakamoto, Konosuke Moritani, Shunsuke Tsukamoto, Yukihide Kanemitsu

**Affiliations:** Colorectal Surgery Division, National Cancer Center Hospital, 5-1-1 Tsukiji, Chuo-ku, Tokyo, 1040045 Japan

**Keywords:** Controlling nutritional status (CONUT) score, Comorbidity index, Older, Colorectal cancer

## Abstract

**Background:**

Assessment of preoperative general condition to predict postoperative outcomes is important, particularly in older patients who typically suffer from various comorbidities and exhibit impaired functional status. In addition to various indices such as Charlson Comorbidity Index (CCI), National Institute on Aging and National Cancer Institute Comorbidity Index (NIA/NCI), Adult Comorbidity Evaluation-27 (ACE-27), and American Society of Anesthesiologists Physical Status classification (ASA-PS), controlling nutritional status (CONUT) score is recently gaining attention as a tool to evaluate the general condition of patients from a nutritional perspective. However, the utility of these indices in older patients with colorectal cancer has not been compared.

**Methods:**

The study population comprised 830 patients with Stage I - IV colorectal cancer aged 75 years or older who underwent surgery at the National Cancer Center Hospital from January 2000 to December 2014. Associations of each index with overall survival (OS) (long-term outcome) and postoperative complications (short-term outcome) were examined.

**Results:**

For the three indices with the highest Akaike information criterion values (i.e., CONUT score, CCI and ACE-27), but not the remaining indices (NIA/NCI and ASA-PS), OS significantly worsened as general condition scores decreased, after adjusting for known prognostic factors. In contrast, for postoperative complications, only CONUT score was identified as a predictive factor (≥4 versus 0–3; odds ratio: 1.90; 95% CI: 1.13–3.13; *P* = 0.016).

**Conclusion:**

For older patients with colorectal cancer, only CONUT score was a predictive factor of both long-term and short-term outcomes after surgery, suggesting that CONUT score is a useful preoperative risk assessment index.

## Background

As older populations increase globally, colorectal cancer surgery is expected to become more common. Older patients typically suffer from several comorbidities and exhibit impaired functional status, which lead to higher postoperative morbidity and mortality compared with younger patients [1, 2]. Thus, assessing the preoperative general condition of older patients in particular is important for predicting postoperative short-term and long-term outcomes.

Various risk assessment indices have been used to evaluate the general condition of patients, including American Society of Anesthesiologists Physical Status classification (ASA-PS) [3], which assesses physical status, and Charlson Comorbidity Index (CCI) [4], National Institute on Aging (NIA) and National Cancer Institute (NCI) Comorbidity Index (NIA/NCI) [5], and Adult Comorbidity Evaluation-27 (ACE-27) [6], which are used to assess comorbidities. For colorectal cancer, ASA-PS and CCI reportedly predict postoperative complications [7], and CCI, NIA/NCI, and ACE-27 predict overall survival (OS) [8, 9]. Poor general condition is associated with increased postoperative complications and decreased survival after surgery.

Controlling nutritional status (CONUT) score [10] is another index that evaluates general condition from a nutritional perspective. CONUT score is calculated from serum albumin (indicator of protein reserves), total cholesterol concentration (caloric depletion parameter), and total peripheral lymphocyte counts (indicator of weak immune defense due to undernutrition) [10]. Recently, CONUT score has been reported to be a prognostic factor for survival in patients with different types of cancer, including colorectal cancer [11, 12], gastric cancer [13–15], esophageal cancer [15–17], hepatocellular carcinoma [18], intrahepatic cholangiocarcinoma [19], and lung cancer [20]. However, the relationship between CONUT score and postoperative complications in cancer patients remains controversial [11, 13, 16, 19].

Little is known about the relationships between risk assessment indices that evaluate general condition and short-term and long-term outcomes in older patients with cancer. Accordingly, this study aimed to examine the association of risk assessment indices with both OS (long-term outcomes) and postoperative complications (short-term outcomes) in older patients with colorectal cancer.

## Methods

### Study population

Subjects of this retrospective study were patients with colorectal cancer aged 75 years or older who were treated at the National Cancer Center Hospital from January 2000 to December 2014. Patients with Stage 0 cancer, patients who did not undergo surgery due to unresectable Stage IV cancer, and patients for whom CONUT scores could not be calculated due to insufficient data were excluded. This retrospective study was approved by the Institutional Review Board (IRB) of the National Cancer Center Hospital (IRB code: 2017–437).

### Data collection

The following parameters were retrospectively assessed using medical records: age, sex, body mass index (BMI) (≥25 versus < 25), primary tumor site (colon versus rectum), presence of lymph node metastasis, carcinoembryonic antigen (CEA) (≤5 versus > 5), carbohydrate antigen 19–9 (CA19–9) (≤37 versus > 37), stage according to the Union for International Cancer Control TNM classification (8th edition) [21], and postoperative complications. Postoperative complications in this study were defined as a morbidity that occurred within duration of postoperative hospital stay or within 30 days after surgery, and as a morbidity with a Clavien-Dindo classification ≥II (See Additional file 1: Table S1 for a list of complication definitions) [22].

### Indices of general condition: CONUT score, ASA-PS, CCI, NIA/NCI, ACE-27

CONUT scores were calculated using data for serum albumin, total cholesterol concentrations, and total peripheral lymphocyte counts based on a previous report that used preoperative serum samples [10]. Albumin concentrations ≥3.5, 3.0–3.49, 2.5–2.99, and < 2.5 g/dL were scored as 0, 2, 4, and 6 points, respectively; (2) total lymphocyte counts ≥1600, 1200–1599, 800–1199, and < 800/mm3 were scored as 0, 1, 2, and 3 points, respectively; and (3) total cholesterol concentrations ≥180, 140–179, 100–139, and < 100 mg/ dL were scored as 0, 1, 2, and 3 points, respectively. The CONUT score was defined as the sum of (1), (2), and (3). Comorbidity information was obtained from medical records up to the date of surgery. Information was obtained from physician notes, anesthesia notes (ASA-PS), nursing notes, and discharge summaries. All comorbid conditions were then indexed according to the CCI, ACE-27, and NIA/NCI, and relative scores were obtained.

For CONUT score, patients were divided into three groups: scores of 0, 1 / 2, 3 / ≥4. For ASA-PS, patients were divided into three groups: scores of 1 / 2 / 3. For CCI, patients were divided into three groups based on the sum of weighted comorbidities: 2 / 3 / ≥4. For NIA/NCI, patients were divided into three groups corresponding to the total number of comorbidities: 0, 1 / 2, 3 / ≥4. For ACE-27, patients were divided into four groups: none, mild, moderate, or severe comorbidity.

### Statistical analysis

Data are presented as numbers of patients, ratios (%), hazard ratios (HRs), or odds ratios (ORs) and 95% confidence intervals (CIs). OS was defined as the interval between the date of diagnosis of colorectal cancer and the date of death from all causes. Survivors were censored as of the date of data cut-off (April 2018). The Kaplan-Meier method was used to estimate OS. Differences in survival were assessed with the log-rank test. Models for Cox proportional hazards were constructed separately for the five indices and were used to calculate HRs and 95% CIs. HRs adjusted for sex, BMI, lymph node metastasis, stage, CEA, and CA19–9, all of which were reported to be significant covariates in the previous studies [8, 11], were also calculated. BMI was included in the analysis as a categorical parameter (≥25 versus < 25). To estimate the goodness-of-fit of each index based on Cox regression survival analysis, Akaike Information Criterion (AIC) values were compared between the five indices. AIC was calculated as follows: AIC = − 2 log maximum likelihood + 2 x (number of parameters in the model). Smaller AIC values represent better optimistic prognostic stratification. Logistic regression analysis models were used to calculate ORs and 95% CIs for postoperative complications in each index. *P* <  0.05 was considered statistically significant. All statistical analyses were performed using the JMP14 software program (SAS Institute Japan Ltd., Tokyo, Japan).

## Results

### Study cohort characteristics

**Fig. 1:**
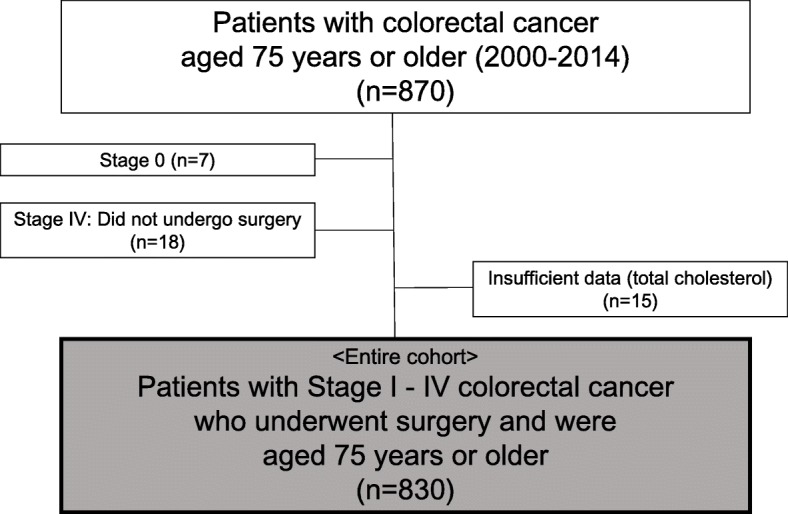
Study cohort. After excluding patients with Stage 0 cancer (*n* = 7), patients who did not undergo surgery (*n* = 18), and patients for whom CONUT scores could not be calculated due to insufficient data (*n* = 15) from an initial pool of 870 patients with colorectal cancer aged 75 years or older, the final study population consisted of 830 patients

Details of the study cohort are summarized in Fig. 1. Between 2000 and 2014, a total of 870 patients with colorectal cancer aged 75 years or older were treated at the National Cancer Center Hospital. Of these, we excluded 7 patients with Stage 0 cancer, 18 patients who did not undergo surgery due to unresectable stage IV cancer, and 15 patients for whom CONUT scores could not be calculated due to insufficient data (all were missing data for total cholesterol concentration). The final study population consisted of 830 patients with stage I - IV colorectal cancer who underwent surgery and were aged 75 years or older. Patient characteristics stratified by CONUT category are summarized in Table 1. For CONUT scores, the number of patients with scores of 0, 1 / 2, 3 / ≥4 were 508 (61%), 249 (30%), and 73 (9%), respectively. The median patient age was 78 years (range, 75–94 years), and 470 patients (57%) were male and 360 (43%) were female. Of the 830 patients, 653 (79%) had a tumor in the colon, 482 (58%) had stage I or II colorectal cancer, and 348 (42%) had stage III or IV colorectal cancer. Patients with higher stage were also the patients with higher CONUT score (*p* = 0.045).

**Table 1:** Patient characteristics (*n* = 830)

	Total (*n* = 830)	CONUT score			*p* value
		0/1 (*n* = 508, 61%)	2/3 (*n* = 249, 30%)	≥4 (*n* = 73, 9%)	
Age, years					0.022
median (range)	78 (75–94)	78 (75–92)	79 (75–94)	80 (75–88)	
Sex					0.115
Male	470 (57%)	277 (55%)	144 (58%)	49 (67%)	
Female	360 (43%)	231 (45%)	105 (42%)	24 (33%)	
BMI					0.055
< 25	681 (82%)	408 (80%)	206 (83%)	67 (92%)	
≥ 25	149 (18%)	100 (20%)	43 (17%)	6 (8%)	
Primary tumor site					0.380
Colon	653 (79%)	398 (78%)	193 (78%)	62 (85%)	
Rectum	177 (21%)	110 (22%)	56 (22%)	11 (15%)	
Stage					0.045
I	224 (27%)	152 (30%)	59 (24%)	13 (18%)	
II	258 (31%)	152 (30%)	77 (32%)	29 (40%)	
III	258 (31%)	159 (31%)	80 (32%)	19 (26%)	
IV	90 (11%)	45 (9%)	33 (13%)	12 (16%)	
ASA-PS					< 0.001
1	98 (12%)	71 (14%)	22 (9%)	5 (7%)	
2	571 (69%)	360 (71%)	175 (70%)	36 (49%)	
3	161 (19%)	77 (15%)	52 (21%)	32 (44%)	
CCI					0.001
2	532 (64%)	345 (68%)	151 (61%)	36 (49%)	
3	156 (19%)	95 (19%)	48 (19%)	13 (18%)	
≥ 4	142 (17%)	68 (13%)	50 (20%)	24 (33%)	
NIA/NCI					0.001
0/1	376 (45%)	246 (48%)	100 (40%)	30 (41%)	
2/3	381 (46%)	233 (46%)	119 (48%)	29 (40%)	
≥ 4	73 (9%)	29 (6%)	30 (12%)	14 (19%)	
ACE-27					0.130
Normal	0	0	0	0	
Mild	0	0	0	0	
Moderate	487 (59%)	310 (61%)	133 (53%)	44 (60%)	
Severe	343 (41%)	198 (39%)	116 (47%)	29 (40%)	

*CONUT* Controlling Nutritional Status, *BMI* body mass index, *ASA-PS* American Society of Anesthesiologists Physical Status classification, *CCI* Charlson Comorbidity Index, *NIA/NCI* National Institute on Aging and National Cancer Institute Comorbidity Index, *ACE-27* Adult Comorbidity Evaluation-27

A majority of patients scored 2 (*n* = 571, 69%) on the ASA-PS, 2 on the CCI (*n* = 532, 64%), and had 2 / 3 comorbidities (*n* = 381, 46%) on the NIA/NCI. For the ACE-27, most patients were classified in the moderate group (*n* = 487, 59%), with the remainder of patients classified in the severe group.

### Long-term outcomes classified by each index

**Fig. 2:**
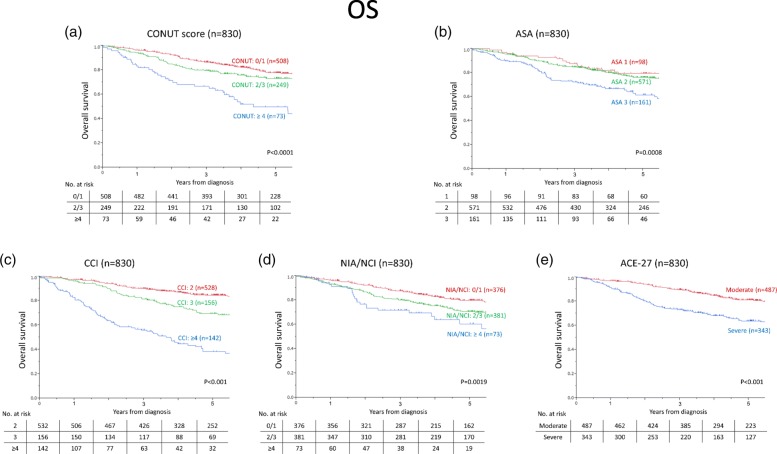
Overall survival curves in patients grouped by (**a**) controlling nutritional status (CONUT) score, (**b**) American Society of Anesthesiologists Physical Status classification (ASA-PS), (**c**) Charlson Comorbidity Index (CCI), (**d**) National Institute on Aging and National Cancer Institute Comorbidity Index (NIA/NCI), and (**e**) Adult Comorbidity Evaluation-27 (ACE-27)

Figure 2 shows OS curves for each index. Five-year OS rates in patients with CONUT scores of 0, 1 / 2,3 / ≥4 were 77.7, 73.2, and 49.7%, respectively (*p* <  0.0001). For the ASA-PS, five-year OS rates for scores of 1 / 2 / 3 were 79.4, 76.0, and 61.5%, respectively (*p* = 0.0008). For the CCI, five-year OS rates grouped by scores of 2 / 3 / ≥4 were 84.1, 69.4, and 38.4%, respectively (*p* <  0.001). For the NIA/NCI, five-year OS rates grouped by 0, 1 / 2, 3 / ≥4 were 79.8, 70.8, and 60.4%, respectively (*p* = 0.0019). For the ACE-27, five-year OS rates grouped by moderate and severe were 81.1 and 63.7%, respectively (*p* <  0.001).

### Associations between each index and long-term outcomes

Cox proportional hazards models were constructed for the five indices, and HRs of OS in each index are shown in Table 2. HRs adjusted for sex, BMI, lymph node metastasis, Stage, CEA, and CA19–9, were also investigated and shown in Table 2. For CONUT score, CCI, and ACE-27, as scores worsened, OS also significantly worsened, when adjusting for the above-mentioned covariates (CONUT score: 2/3 versus 0/1, HR = 1.35, 95% CI: 1.00–1.81, ≥4 versus 0/1, HR = 2.24, 95% CI: 1.48–3.30, ≥4 versus 2/3, HR = 1.65, 95% CI: 1.07–2.51; CCI: 3 versus 2, HR = 1.62, 95% CI: 1.14–2.28, ≥4 versus 2, HR = 3.05, 95% CI: 2.20–4.24; ACE-27: severe versus moderate, HR = 1.80, 95% CI: 1.37–2.37). In contrast, for NIA/NCI and ASA-PS, OS did not significantly worsen even when general condition worsened, when adjusting for known prognostic factors. Among the covariates used in each multivariate analysis, BMI, lymph node metastasis, Stage, CEA, and CA19–9, were also independent prognostic factors (data not shown).

**Table 2:** Association of each index with overall survival

Variable	Unadjusted			Adjusted^a^		
	HR	95% CI	*p* value	HR	95% CI	*p* value
CONUT score						
0/1	Ref	–	–	Ref	–	–
2/3	1.38	1.03–1.85	0.033	1.35	1.00–1.81	0.048
≥ 4	2.70	1.82–3.91	< 0.001	2.24	1.48–3.30	< 0.001
ASA-PS						
1	Ref	–	–	Ref	–	–
2	1.23	0.81–1.94	0.34	1.23	0.81–1.95	0.337
3	2.08	1.31–3.42	0.002	2.24	1.39–3.70	0.001
CCI						
2	Ref	–	–	Ref	–	–
3	1.91	1.35–2.67	< 0.001	1.62	1.14–2.28	0.008
≥ 4	4.98	3.68–6.72	< 0.001	3.05	2.20–4.24	< 0.001
NIA/NCI						
0/1	Ref	–	–	Ref	–	–
2/3	1.49	1.13–1.98	0.005	1.29	0.97–1.72	0.085
≥ 4	2.03	1.25–3.16	0.005	1.70	1.04–2.69	0.036
ACE-27						
Moderate	Ref	–	–	Ref	–	–
Severe	2.14	1.64–2.79	< 0.001	1.80	1.37–2.37	< 0.001

*CONUT* Controlling Nutritional Status, *ASA-PS* American Society of Anesthesiologists Physical Status classification, *CCI* Charlson Comorbidity Index, *NIA/NCI* National Institute on Aging and National Cancer Institute Comorbidity Index, *ACE-27* Adult Comorbidity Evaluation-27, *HR* hazard ratio, *CI* confidence interval

^a^Hazard ratios adjusted for sex, BMI, lymph node metastasis, Stage, CEA, and CA19–9

### AIC of each index model

AIC was used as a parameter for goodness-of-fit, with lower AIC values indicative of goodness-of-fit. AIC values of each index were 2764.52 for CONUT score, 2774.59 for ASA-PS, 2690.13 for CCI, 2775.19 for NIA/NCI, and 2753.13 for ACE-27. According to this comparison, CCI had the best goodness-of-fit, followed by ACE-27 and CONUT score.

### Postoperative complications

The total number of patients with postoperative complications of Clavien-Dindo classification ≥II was 216 (26% of total patients). Of these, there were 141, 55, 15, 2, and 3 patients with Clavien-Dindo classification II, IIIa, IIIb, IVa, and V, respectively. The most common complication was ileus or intestinal obstruction, which accounted for 63 patients (7.6%), followed by surgical site infection (*n* = 36; 4.3%), urinary tract infection (*n* = 34; 4.1%), pneumonia / respiratory failure (*n* = 30; 3.6%), wound dehiscence (*n* = 26; 3.1%), other infections (*n* = 12; 1.4%), anastomotic leakage (*n* = 9; 1.1%), vascular events (*n* = 8; 1.0%), intra-abdominal abscess (*n* = 6; 0.7%), and others (*n* = 21; 2.5%). Other infections included pseudomembranous colitis, cholangitis, parotitis, and catheter infection. Vascular events included cerebral infarction, angina attack, pulmonary embolism, arteriosclerosis obliterans, and acute peripheral artery occlusive disease. The “other” category included anastomotic bleeding, arrhythmia, peptic ulcer, urinary retention, drug eruption, convulsion, pneumothorax, gastrointestinal perforation, chylorrhea, ascites, and facial nerve paralysis. Three complications of Clavien-Dindo classification V consisted of one pneumonia / respiratory failure case and two vascular events.

### Associations between each index and postoperative complications

Univariate and multivariate logistic regression analyses to assess associations of each index with postoperative complications are shown in Table 3. Univariate analysis showed that sex (*p* = 0.005), tumor location (*p* = 0.003), and CONUT score (*p* = 0.015), but not BMI (*p* = 0.648), were significantly associated with postoperative complications. There was no significant association between the four comorbidity indices and postoperative complications.

**Table 3:** Univariate and multivariate logistic regression analyses of correlations of each index with postoperative complications (Clavien Dindo ≥2)

Variable	Objective variable	Control	Univariate analysis			Multivariate analysis		
			OR	95% CI	*p* value	OR	95% CI	*p* value
Age	≥85	75–84	1.66	0.63–1.66	0.894			
Sex	male	female	1.59	1.15–2.19	0.005	1.53	1.10–2.12	0.010
BMI	≥25	< 25	1.10	0.73–1.62	0.648	1.19	0.79–1.78	0.397
Tumor location	rectum	colon	1.75	1.22–2.49	0.003	1.79	1.24–2.56	0.002
CONUT score	≥4	0–3	1.88	1.13–3.09	0.015	1.93	1.15–3.20	0.013
ASA-PS	3	1/2	0.93	0.62–1.37	0.703			
CCI	≥4	< 4	0.84	0.54–1.26	0.402			
NIA/NCI	≥4	0–3	0.78	0.43–1.36	0.395			
ACE-27	Severe	Moderate	0.76	0.55–1.05	0.098			

*BMI* body mass index, *CONUT* Controlling Nutritional Status, *ASA-PS* American Society of Anesthesiologists Physical Status classification, *CCI* Charlson Comorbidity Index, *NIA/NCI* National Institute on Aging and National Cancer Institute Comorbidity Index, *ACE-27* Adult Comorbidity Evaluation-27, *OR* odds ratio, *CI* confidence interval

Multivariate analysis showed that CONUT score ≥ 4 was an independent predictor of postoperative complications (OR = 1.93; 95% CI (1.15–3.20); *p* = 0.013), indicating that, among the five indices, only CONUT score was an independent predictor of short-term outcomes.

## Discussion

This study had two notable points. First, we focused on older patients with colorectal cancer who typically have several comorbidities and impaired functional status that may lead to higher operative risk. Second, we included CONUT score as an index to evaluate the relationship of a patient’s general condition with OS and postoperative complications. Through these new approaches, we demonstrated that among the five indices evaluated (CONUT score, ASA-PS, CCI, NIA/NCI, ACE-27), only CONUT score was a significant prognostic factor of both OS (long-term outcomes) and postoperative complications (short-term outcomes) in older patients with colorectal cancer. This suggests that CONUT score may be useful as a preoperative risk assessment index in this patient population.

In terms of long-term outcomes, for CONUT score, CCI, and ACE-27, but not ASA-PS and NIA/NCI, as scores for general condition worsened, OS became significantly worse as well. Moreover, an assessment of AIC revealed that these three indices had better AIC values than those of ASA-PS and NIA/NCI. Taken together, these results suggest that, among the five indices, CONUT score, CCI, and ACE-27 were good models for predicting OS of older patients with colorectal cancer. Our results are compatible with previous studies reporting that CONUT score [11], CCI [8, 9], and ACE-27 [8, 9] predict OS of patients with colorectal cancer, although not specifically older patients [8, 9]. Despite NIA/NCI not being a predictor of OS in our study, it was a predictor in other studies involving patients with colorectal cancer [8, 9].

It is not surprising that CONUT score is a prognostic factor for OS in various types of cancers [11–20] because each of its three components reflects cancer progression. Serum albumin is a marker of nutritional status and reportedly correlates with tumor necrosis, as pro-inflammatory cytokines reduce albumin synthesis [23]. Total cholesterol concentration has been reported to correlate with tumor progression, as tumor tissue reduces plasma cholesterol concentration and caloric intake [24]. Finally, total lymphocyte counts reflect immunological status, and a low peripheral lymphocyte count is associated with worse prognosis in several cancers due to insufficient host immune response to cancer cells [25, 26].

Despite the above, the utility of CONUT score for evaluating postoperative complications in patients with cancer remains controversial [11, 13, 16, 19]. In the present study, we revealed that, among the five indices, only CONUT score was an independent predictor of short-term outcomes. CONUT score has an advantage over the other indices due to its calculation method. Whereas the CCI requires 19 variables, NIA/NCI requires 24 variables, and ACE-27 requires 27 variables, CONUT score can be easily calculated using only three routinely measured parameters. Thus, CONUT score is an easy and convenient tool for predicting complications, which is not surprising because poor preoperative nutritional status reportedly correlates with the incidence of postoperative complications [27].

Some studies have reported that nutritional intervention for preoperative malnutrition contributes to a reduction of postoperative complications, reduction of length of hospital stay, and reduction of medical costs [28–31]. Our results support nutritional intervention for high CONUT score groups. CONUT score can be an indicator for the need to initiate nutritional intervention and can also serve as a scoring system to evaluate the therapeutic effects of the intervention. Furthermore, since CONUT score reflects both short-term and long-term outcomes, it can impact surgical treatment strategies and thus be used for stratification in randomized clinical studies of older patients with cancer.

This study has limitations worth noting. First, this study was retrospective in design and included patients from a single institution, although the sample size was much larger compared to those of previous studies. Second, although patients underwent various surgical procedures, with more invasive surgical procedures leading to higher mortality and morbidity, we did not account for this in the present study. Our findings warrant further consideration and validation in a larger series of older patients with colorectal cancer.

## Conclusions

The general condition of patients with colorectal cancer impacts their survival and postoperative complications and thus should be considered in cancer management. We demonstrated that, among five indices which evaluate general condition (CONUT score, ASA-PS, CCI, NIA/NCI, ACE-27), only CONUT score was a significant prognostic factor of both OS (long-term outcomes) and postoperative complications (short-term outcomes) in older patients with colorectal cancer. Our findings suggest that CONUT score is useful not only for assessing nutritional status, but can also be used as a preoperative risk assessment index in older patients with colorectal cancer.

## Supplementary information

**Additional file 1: Table S1.** A list of complication definitions.

## Data Availability

The datasets used or analysed during the current study are available from the corresponding author on reasonable request.

## Abbreviations

ACE-27: Adult Comorbidity Evaluation-27
AIC: Akaike information criterion
ASA-PS: American Society of Anesthesiologists Physical Status classification
BMI: Body mass index
CEA: Carcinoembryonic antigen
CA19–9: Carbohydrate antigen 19–9
CCI: Charlson Comorbidity Index
CSS: Cancer-specific survival
CONUT: Controlling nutritional status
NIA/NCI: National Institute on Aging and National Cancer Institute Comorbidity Index
OS: Overall survival
