# Supplementary material for: Controlling nutritional status (CONUT) score as a preoperative risk assessment index for older patients with colorectal cancer
Source: BMC Cancer. 2019 Nov 6;19:946. doi: 10.1186/s12885-019-6218-8 (PMC6833132; doi:10.1186/s12885-019-6218-8)
Supplement: Supplementary file 1 — Additional file 1: Table S1. A list of complication definitions. [file 12885_2019_6218_MOESM1_ESM.docx]

Table S1. A list of complication definitions

| Complication | Definition |
| --- | --- |
| Surgical site infection | an infection in the area of the body affected by surgery and included superficial incisional, deep incisional infection |
| Pneumonia | the presence of new infiltrates on chest radiography |
| Intestinal obstruction | a morbidity requiring any intervention such as decompression by nasal gastric tube, endoscopic, radiological, or surgical intervention |
| Anastomotic leakage | the various signs of clinical leakage such as emission of dirty fluid from drain, and/or a radiographically apparent leakage confirmed by CT |
| Vascular event | the presence of any cardiac disease such as arrhythmia and ischemic heart disease, and the presence of any thrombosis |
